# 

**Published:** 1860-05

**Authors:** 


NORTH AMERICAN

### MEDICO-CHIRURGICAL REVIEW.

Vol. IV.] MAY, 1860. [No. 3.

### CONTENTS.

### ANALYTICAL AND CRITICAL REVIEWS.

ART. PAGE

I.—Medical Literature of Philadelphia .......... 385

II.—1. A System of Dental Surgery; by JOHN TOMES, F.R.S.—2. A Practical Treatise on Operative Dentistry; by PROF. J. TAFT .......... 414

III.—Blood Disease. By J. VAUGHAN HUGHES, M.D. .......... 427

IV.—Proceedings and Debates of the Third National Quarantine and Sanitary Convention, held April, 1859 .......... 431

V.—Elements of Medical Jurisprudence. By THEODRIC ROMEYN BECK, M.D., LL.D., and JOHN B. BECK, M.D .......... 433

VI.—Introductory Lectures and Addresses on Medical Subjects. By GEORGE B. WOOD, M.D., LL.D .......... 443

VII.—Lectures on the Diseases of Infancy and Childhood. By C. WEST, M.D.. 445

VIII.—Transactions of the Medical Society of the State of Pennsylvania. 446

IX.—Clinical Lectures on the Diseases of Women and Children. By GUNNING S. BEDFORD, M.D .......... 448

X.—Clinical Lectures on the Principles and Practice of Medicine. By JOHN HUGHES BENNETT, M.D .......... 449

XI.—A Clinical Memoir on Strangulated Hernia. By GEORGE MACILWAIN... 450

XII.—An Epitome of Braithwaite's Retrospect. By WALTER S. WELLS, M.D.. 452

### ORIGINAL COMMUNICATIONS.

I.—Cases from the Surgical Clinic of the Philadelphia Hospital.—Service of PROF. GROSS. By EMIL FISCHER, M.D .......... 453

II.—Account of Remarkable Ovarian Tumors. By JOSEPH H. WYTHES, M.D., 478

III.—A Case of Aneurism of the Femoral Artery cured by Mechanical Compression. By W. A. CLAPP, M.D .......... 484

IV.—A Case of Removal of the Patella of the Left Knee. By O. B. KNODE, M.D.. 486

V.—Proceedings of the Pathological Society of Philadelphia .......... 488

### BI-MONTHLY ABSTRACTS.

#### SURGERY. BY S. W. GROSS, M.D.

On Scalds of the Larynx.—Selections from a Report on Ovariotomy.—On Polyps of the Œsophagus.—A Historical and Critical Examination of the Operation of Tracheotomy in Croup.—On Tibio-tarsal Amputation.—On Tic Douloureux, with a New Operation for its Cure .......... 501

#### PATHOLOGY AND PRACTICE OF MEDICINE. BY RICHARD J. DUNGLISON, M.D.

Congenital Inequality of the Two Sides of the Body.—Coagulation of the Blood in the Venous System during Life.—A Case Illustrating the Pathology of the Cerebellum.—Phthisis of the French Millstone-makers.—Small-pox in Scotland.—Œsophageal Fistula.—Virchow's Views on Cellular Pathology .......... 509

OBSTETRICS, AND DISEASES OF WOMEN AND CHILDREN. BY WM. B. ATKINSON, M.D.

PAGE

Post-Partum Hemorrhage.—Aids in the Treatment of Impeded Parturition.—Influence of the Mother's Mind on the Fœtus.—The Uterus and its Inflexions.—On the Difficulty of Diagnosis between Pregnancy and Tumors of the Abdomen.—Practical Remarks on Fœtal Auscultation.—The Mental Peculiarities and Disorders of Childhood.—Iodide of Potassium in Diseases of the Brain in Children........ 520

PHYSIOLOGY. BY S. WEIR MITCHELL, M.D.

Remarks on a Critique of Dr. Brinton's on the Pancreas.—Notes on Experiments on the Sympathetic Nerve of a Beheaded Woman.—On the Existence of Certain Smooth Muscular Fibres in the Orbit of Man and Mammals.—Urea in the Fluids of the Thoracic Duct........ 528

MATERIA MEDICA AND THERAPEUTICS; MEDICAL CHEMISTRY; TOXICOLOGY AND LEGAL MEDICINE. BY EMIL FISCHER, M.D.

*Materia Medica and Therapeutics:* Medicinal Properties of Globularia Alypum, L.—Notes on the Cornelian Cherry.—External Use of Cyanide of Potassium in Neuralgia.—Iodide of the Chloride of Mercury in the Treatment of Skin Diseases.—Chloride Lotions in Variola.—Pills of Carbonate of Ammonia in Chronic Bronchitis.—Pencils of Tannin in Affections of the Uterus.—Treatment of Burns by Local Application of Distilled Cherry-Laurel Water.—Treatment of Excessive Perspiration of the Feet.—Treatment of Ascarides.—Infusion of Coffee in Strangulated Hernia.—Muriate of Ammonia in Nervous Cephalalgia.—A New Mode of Preparing and Applying Chloride of Zinc.—Natural History, etc., of the Sanguinaria Canadensis.—Inhalation of Chloroform.—Remarks on Propylamin.—Hypophosphites of Soda and of Lime in the Treatment of Phthisis.—Saccharated Lime.—Vesicating Principle of Lytta Vittata........ 534

*Toxicology and Legal Medicine:* Remarks on Nitro-Benzine.—On the Detection of Phosphorus.—Arsenical Fly-Papers.—Arsenical Poisoning by Paper.—Detection of Blood-Stains........ 548

BI-MONTHLY PERISCOPE.

SURGERY.

Cases of Amputations, with Acupressure.—Intermittent Lameness in the Horse from Emboli, or Obliteration of the Iliac Arteries........ 552

OBSTETRICS.

Cephalic Version of the Fœtus in the Uterus by External Version........ 559

EDITORS' TABLE.—Schiller as a Physician.—The State Medical Society of Pennsylvania.—Changes in Medical Schools.—Medical Students and Graduates in the United States during the Session of 1859–60.—Students in Philadelphia.—Students in London and Paris.—Dental Colleges.—Philadelphia College of Pharmacy.—Man, Moral and Physical.—Medical Journals.—Prize offered to the Medical Students of the United States.—Prizes Awarded by the Academy of Medicine of Paris, for 1859.—Prizes of the Paris Academy of Medicine, for 1862.—Marriages of Consanguinity.—American Medical Literature Abroad.—Dixon on the Eye, and Althaus on Electricity.—Suicide in Massachusetts.—Portrait of Professor Wood.—Dr. W. T. G. Morton, of Boston.—Veterinary Science in Europe.—Chair of Surgery in the University of Glasgow.—Acupressure.—Just Sentiments.—Parisian Doings.—New Orleans Medical News and Hospital Gazette.—To Correspondents........ 563

NECROLOGICAL NOTICES.—Charles Frick, M.D.—Pitman Clemens Spencer, M.D.—Robert Bentley Todd, M.D., F.R.S........ 573

BI-MONTHLY BIBLIOGRAPHICAL RECORD........ 574

